# Bis[*N*-(2-hy­droxy­eth­yl)-*N*-propyl­dithio­carbamato-κ^2^ *S*,*S*′]bis­(4-{[(pyridin-4-yl­methyl­idene)hydrazinyl­idene]meth­yl}pyridine-κ*N* ^1^)cadmium

**DOI:** 10.1107/S1600536811004508

**Published:** 2011-02-12

**Authors:** Grant A. Broker, Edward R. T. Tiekink

**Affiliations:** a 5959 FM 1960 Road West, Houston, Texas 77069, USA; b Department of Chemistry, University of Malaya, 50603 Kuala Lumpur, Malaysia

## Abstract

The complete mol­ecule of the title compound, [Cd(C_6_H_12_NOS_2_)_2_(C_12_H_10_N_4_)_2_], is generated by crystallographic inversion symmetry. The distorted octa­hedral *trans*-N_2_S_4_ donor set for the Cd^2+^ ion is defined by two symmetrically *S*,*S*′-chelating dithio­carbamate anions and two pyridine N atoms derived from two monodentate 4-pyridine­aldazine (or 4-{[(pyridin-4-yl­methyl­idene)hydrazinyl­idene}meth­yl]pyridine) mol­ecules [dihedral angle between the aromatic rings = 17.33 (8)°]. In the crystal, mol­ecules are connected into a supra­molecular chain *via* O—H⋯N hydrogen bonds involving the 4-pyridine­aldazine N atoms not involved in coordination to cadmium. Weak C—H⋯O and C—H⋯N links consolidate the packing.

## Related literature

For background to supra­molecular coordination polymers of zinc-triad 1,1-dithiol­ates, see: Tiekink (2003 ▶). For the use of steric effects to control supra­molecular aggregation patterns, see: Chen *et al.* (2006 ▶). For structural studies on hydroxyl-substituted dithio­carbamate ligands, see Benson *et al.* (2007 ▶); Song & Tiekink (2009 ▶).
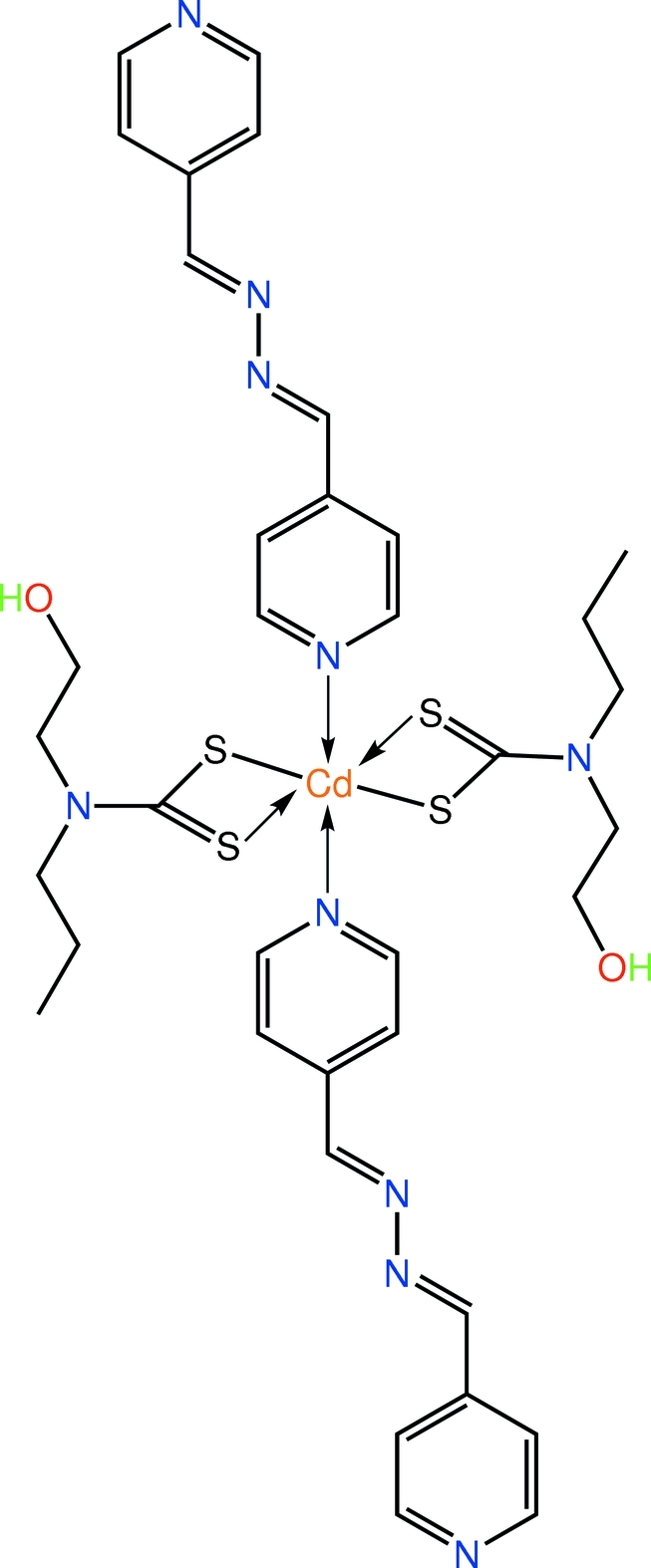


## Experimental

### 

#### Crystal data

[Cd(C_6_H_12_NOS_2_)_2_(C_12_H_10_N_4_)_2_]
*M* *_r_* = 889.45
Triclinic, 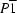

*a* = 8.532 (3) Å
*b* = 10.951 (4) Å
*c* = 11.184 (5) Å
α = 79.59 (3)°
β = 88.06 (3)°
γ = 78.23 (2)°
*V* = 1006.2 (7) Å^3^
*Z* = 1
Mo *K*α radiation
μ = 0.80 mm^−1^
*T* = 98 K
0.25 × 0.16 × 0.04 mm

#### Data collection

Rigaku AFC12/SATURN724 CCD diffractometer
Absorption correction: multi-scan (*ABSCOR*; Higashi, 1995 ▶) *T*_min_ = 0.719, *T*_max_ = 1
10677 measured reflections
4150 independent reflections
4009 reflections with *I* > 2σ(*I*)
*R*_int_ = 0.023

#### Refinement

*R*[*F* ^2^ > 2σ(*F* ^2^)] = 0.025
*wR*(*F* ^2^) = 0.062
*S* = 1.08
4150 reflections
245 parameters
1 restraint
H atoms treated by a mixture of independent and constrained refinement
Δρ_max_ = 0.40 e Å^−3^
Δρ_min_ = −0.40 e Å^−3^

### 

Data collection: *CrystalClear* (Molecular Structure Corporation & Rigaku, 2005 ▶); cell refinement: *CrystalClear*; data reduction: *CrystalClear*; program(s) used to solve structure: PATTY in *DIRDIF* (Beurskens *et al.*, 1992 ▶); program(s) used to refine structure: *SHELXL97* (Sheldrick, 2008 ▶); molecular graphics: *ORTEPII* (Johnson, 1976 ▶) and *DIAMOND* (Brandenburg, 2006 ▶); software used to prepare material for publication: *publCIF* (Westrip, 2010 ▶).

## Supplementary Material

Crystal structure: contains datablocks global, I. DOI: 10.1107/S1600536811004508/hb5795sup1.cif

Structure factors: contains datablocks I. DOI: 10.1107/S1600536811004508/hb5795Isup2.hkl

Additional supplementary materials:  crystallographic information; 3D view; checkCIF report

## Figures and Tables

**Table:** 

Cd—S1	2.6379 (10)
Cd—S2	2.6626 (10)
Cd—N2	2.5403 (17)

**Table:** 

S1—Cd—S2	68.83 (3)

**Table 2:** Hydrogen-bond geometry (Å, °)

*D*—H⋯*A*	*D*—H	H⋯*A*	*D*⋯*A*	*D*—H⋯*A*
O1—H1*o*⋯N5^i^	0.84 (2)	1.98 (2)	2.810 (2)	176 (2)
C10—H10⋯O1^ii^	0.95	2.55	3.480 (3)	168
C3—H3a⋯N4^iii^	0.99	2.61	3.369 (3)	134

Symmetry codes: (i) 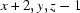; (ii) 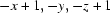; (iii) 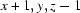.

**Fig. 1.**
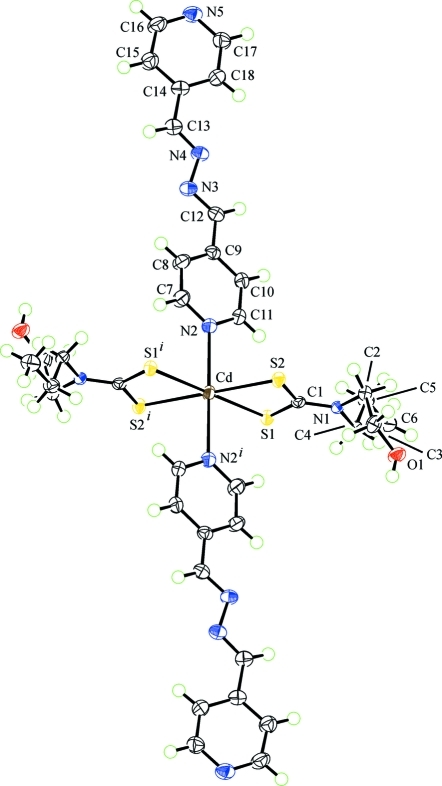
Molecular structure of (I) showing displacement ellipsoids at the 70% probability level. The Cd atom is located on a centre of inversion and i = 1 - x, 1 - y, 1 - z.

**Fig. 2.**
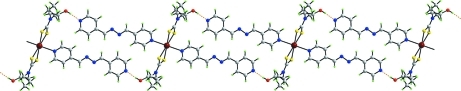
Supramolecular chain in (I) mediated by O–H···N (orange dashed lines) hydrogen bonds. Colour code: Cd, orange; S, yellow; O, red; N, blue; C, grey; and H, green.

**Fig. 3.**
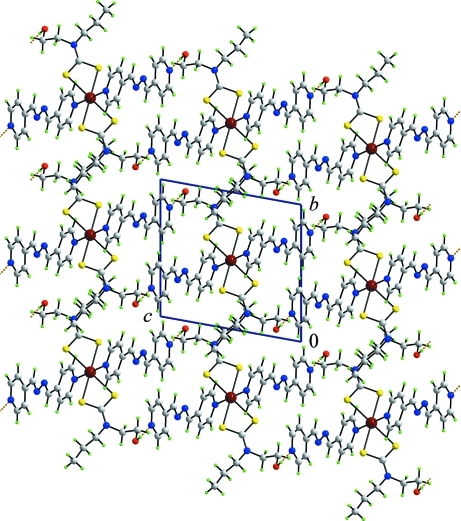
Unit-cell contents in (I) viewed in projection down the a axis.

## supplementary crystallographic information

### Comment

Interest in the title compound, (I), relates to controlling supramolecular aggregation patterns in the zinc-triad 1,1-thiolates (Tiekink, 2003; Chen *et al.*, 2006). With functionalized dithiocarbamate ligands carrying hydrogen bonding potential, smaller aggregates can be linked into 2-D and 3-D architectures (Benson *et al.*, 2007; Song & Tiekink, 2009). In (I), the cadmium atom is located on a centre of inversion and is chelated by symmetrically coordinating dithiocarbamate ligands, Table 1 and Fig. 1. The octahedral N_2_S_4_ donor set is completed by two pyridine-N atoms derived from two monodentate 4-pyridinealdazine ligands.

The monomeric molecules are connected into a supramolecular chain *via* O–H···N hydrogen bonds, Table 2, that lead to the formation of 40-membered [CdSCNC_2_OH···NC_4_N_2_C_4_N]_2_ synthons, Fig. 2. These chains are linked into layers *via* C–H···O interactions, Table 1, which that stack along [1 0 1]; consolidation of these layers into a 3-D array is afforded by C—H···N_azo_ contacts, Table 2 and Fig. 3.

### Experimental

Compound (I) was prepared following the standard literature procedure (Song & Tiekink, 2009) from the reaction of Cd[S_2_CN(CH_2_CH_2_OH)(nPr)]_2_ and 4-[(1*E*)-[(*E*)-2-(pyridin-4-ylmethylidene)hydrazin-1-ylidene]methyl]pyridine (Sigma Aldrich). Yellow plates of (I) were obtained from the slow evaporation of a chloroform/acetonitrile (3/1) solution.

### Refinement

C-bound H-atoms were placed in calculated positions (C–H 0.95–0.99 Å) and were included in the refinement in the riding model approximation with *U*_iso_(H) set to 1.2–1.5*U*_eq_(C). The O-bound H-atom was located in a difference Fourier map and refined with an O–H restraint of 0.84±0.01 Å, and with *U*_iso_(H) = 1.5*U*_eq_(O). The reflection (81 2) was removed from the final refinement owing to poor agreement.

### Crystal data

**Table:**

[Cd(C_6_H_12_NOS_2_)_2_(C_12_H_10_N_4_)_2_]	*Z* = 1
*M**_r_* = 889.45	*F*(000) = 458
Triclinic, *P*1	*D*_x_ = 1.468 Mg m^−^^3^
Hall symbol: -P 1	Mo *K*α radiation, λ = 0.71070 Å
*a* = 8.532 (3) Å	Cell parameters from 3485 reflections
*b* = 10.951 (4) Å	θ = 2.4–30.3°
*c* = 11.184 (5) Å	µ = 0.80 mm^−^^1^
α = 79.59 (3)°	*T* = 98 K
β = 88.06 (3)°	Plate, yellow
γ = 78.23 (2)°	0.25 × 0.16 × 0.04 mm
*V* = 1006.2 (7) Å^3^	

### Data collection

**Table:**

Rigaku AFC12K/SATURN724 CCD diffractometer	4150 independent reflections
Radiation source: fine-focus sealed tube	4009 reflections with *I* > 2σ(*I*)
graphite	*R*_int_ = 0.023
ω scans	θ_max_ = 26.5°, θ_min_ = 2.4°
Absorption correction: multi-scan (*ABSCOR*; Higashi, 1995)	*h* = −10→10
*T*_min_ = 0.719, *T*_max_ = 1	*k* = −13→12
10677 measured reflections	*l* = −14→14

### Refinement

**Table:**

Refinement on *F*^2^	Primary atom site location: structure-invariant direct methods
Least-squares matrix: full	Secondary atom site location: difference Fourier map
*R*[*F*^2^ > 2σ(*F*^2^)] = 0.025	Hydrogen site location: inferred from neighbouring sites
*wR*(*F*^2^) = 0.062	H atoms treated by a mixture of independent and constrained refinement
*S* = 1.08	*w* = 1/[σ^2^(*F*_o_^2^) + (0.0288*P*)^2^ + 0.6008*P*] where *P* = (*F*_o_^2^ + 2*F*_c_^2^)/3
4150 reflections	(Δ/σ)_max_ < 0.001
245 parameters	Δρ_max_ = 0.40 e Å^−^^3^
1 restraint	Δρ_min_ = −0.40 e Å^−^^3^

### Special details

**Table:**

Geometry. All s.u.'s (except the s.u. in the dihedral angle between two l.s. planes) are estimated using the full covariance matrix. The cell s.u.'s are taken into account individually in the estimation of s.u.'s in distances, angles and torsion angles; correlations between s.u.'s in cell parameters are only used when they are defined by crystal symmetry. An approximate (isotropic) treatment of cell s.u.'s is used for estimating s.u.'s involving l.s. planes.
Refinement. Refinement of *F*^2^ against ALL reflections. The weighted *R*-factor *wR* and goodness of fit *S* are based on *F*^2^, conventional *R*-factors *R* are based on *F*, with *F* set to zero for negative *F*^2^. The threshold expression of *F*^2^ > 2σ(*F*^2^) is used only for calculating *R*-factors(gt) *etc*. and is not relevant to the choice of reflections for refinement. *R*-factors based on *F*^2^ are statistically about twice as large as those based on *F*, and *R*- factors based on ALL data will be even larger.

### Fractional atomic coordinates and isotropic or equivalent isotropic displacement parameters (Å^2^)

**Table:**

	*x*	*y*	*z*	*U*_iso_*/*U*_eq_	
Cd	0.5000	0.5000	0.5000	0.01706 (6)	
S1	0.67567 (5)	0.26992 (4)	0.55937 (3)	0.01562 (9)	
S2	0.48740 (5)	0.36286 (4)	0.32801 (4)	0.01587 (9)	
O1	0.88422 (15)	0.05660 (12)	0.17754 (11)	0.0223 (3)	
H1o	0.954 (2)	0.094 (2)	0.1432 (19)	0.033*	
N1	0.67484 (15)	0.13324 (12)	0.38469 (11)	0.0120 (3)	
N2	0.26143 (17)	0.42763 (14)	0.61140 (13)	0.0192 (3)	
N3	−0.25800 (17)	0.36129 (14)	0.82625 (13)	0.0194 (3)	
N4	−0.37162 (17)	0.28924 (14)	0.87834 (12)	0.0191 (3)	
N5	−0.88990 (18)	0.19365 (15)	1.06431 (14)	0.0245 (3)	
C1	0.61822 (18)	0.24449 (15)	0.41986 (14)	0.0135 (3)	
C2	0.62455 (19)	0.10536 (15)	0.26935 (14)	0.0156 (3)	
H2A	0.5139	0.1526	0.2501	0.019*	
H2B	0.6239	0.0138	0.2796	0.019*	
C3	0.7325 (2)	0.14035 (16)	0.16368 (15)	0.0196 (3)	
H3A	0.6824	0.1356	0.0866	0.024*	
H3B	0.7465	0.2285	0.1601	0.024*	
C4	0.79259 (19)	0.03218 (15)	0.45840 (14)	0.0150 (3)	
H4A	0.8604	0.0717	0.5037	0.018*	
H4B	0.8630	−0.0158	0.4033	0.018*	
C5	0.7154 (2)	−0.05962 (16)	0.54826 (15)	0.0185 (3)	
H5A	0.6421	−0.0957	0.5043	0.022*	
H5B	0.6517	−0.0137	0.6080	0.022*	
C6	0.8430 (2)	−0.16666 (17)	0.61485 (17)	0.0239 (4)	
H6A	0.7911	−0.2254	0.6715	0.036*	
H6B	0.9137	−0.1311	0.6602	0.036*	
H6C	0.9059	−0.2121	0.5557	0.036*	
C7	0.1257 (2)	0.50814 (17)	0.63223 (18)	0.0254 (4)	
H7	0.1197	0.5963	0.6049	0.031*	
C8	−0.0057 (2)	0.46933 (17)	0.69130 (17)	0.0238 (4)	
H8	−0.0989	0.5297	0.7041	0.029*	
C9	0.0008 (2)	0.34018 (16)	0.73174 (14)	0.0169 (3)	
C10	0.1403 (2)	0.25663 (16)	0.70970 (16)	0.0203 (3)	
H10	0.1493	0.1679	0.7352	0.024*	
C11	0.2661 (2)	0.30418 (16)	0.65018 (16)	0.0205 (3)	
H11	0.3608	0.2458	0.6362	0.025*	
C12	−0.1326 (2)	0.28869 (16)	0.79403 (14)	0.0177 (3)	
H12	−0.1255	0.1995	0.8107	0.021*	
C13	−0.5012 (2)	0.35972 (17)	0.90688 (15)	0.0200 (3)	
H13	−0.5119	0.4492	0.8929	0.024*	
C14	−0.6338 (2)	0.30118 (17)	0.96168 (14)	0.0188 (3)	
C15	−0.7640 (2)	0.37321 (18)	1.01209 (17)	0.0248 (4)	
H15	−0.7681	0.4604	1.0127	0.030*	
C16	−0.8877 (2)	0.31554 (19)	1.06143 (17)	0.0269 (4)	
H16	−0.9762	0.3659	1.0952	0.032*	
C17	−0.7628 (2)	0.12403 (18)	1.01712 (16)	0.0247 (4)	
H17	−0.7612	0.0367	1.0192	0.030*	
C18	−0.6341 (2)	0.17338 (18)	0.96559 (16)	0.0228 (4)	
H18	−0.5468	0.1206	0.9332	0.027*	

### Atomic displacement parameters (Å^2^)

**Table:**

	*U*^11^	*U*^22^	*U*^33^	*U*^12^	*U*^13^	*U*^23^
Cd	0.01757 (10)	0.01303 (10)	0.02083 (10)	−0.00255 (7)	0.00354 (7)	−0.00493 (7)
S1	0.0170 (2)	0.01481 (19)	0.01487 (19)	−0.00153 (15)	−0.00065 (15)	−0.00389 (15)
S2	0.0170 (2)	0.01234 (19)	0.0169 (2)	−0.00014 (15)	−0.00203 (15)	−0.00179 (15)
O1	0.0183 (6)	0.0224 (6)	0.0251 (6)	−0.0043 (5)	0.0080 (5)	−0.0026 (5)
N1	0.0114 (6)	0.0119 (6)	0.0122 (6)	−0.0014 (5)	−0.0004 (5)	−0.0016 (5)
N2	0.0185 (7)	0.0181 (7)	0.0218 (7)	−0.0050 (6)	0.0034 (6)	−0.0048 (6)
N3	0.0178 (7)	0.0218 (7)	0.0184 (7)	−0.0064 (6)	0.0024 (5)	−0.0007 (6)
N4	0.0188 (7)	0.0228 (7)	0.0161 (7)	−0.0080 (6)	0.0024 (5)	−0.0003 (6)
N5	0.0222 (8)	0.0305 (9)	0.0219 (8)	−0.0094 (7)	0.0047 (6)	−0.0035 (6)
C1	0.0114 (7)	0.0144 (8)	0.0147 (7)	−0.0042 (6)	0.0032 (6)	−0.0014 (6)
C2	0.0165 (8)	0.0145 (8)	0.0158 (8)	−0.0018 (6)	0.0002 (6)	−0.0037 (6)
C3	0.0222 (9)	0.0195 (8)	0.0156 (8)	−0.0012 (7)	0.0027 (6)	−0.0030 (6)
C4	0.0127 (7)	0.0138 (8)	0.0169 (8)	0.0004 (6)	−0.0007 (6)	−0.0020 (6)
C5	0.0167 (8)	0.0168 (8)	0.0210 (8)	−0.0043 (7)	0.0009 (6)	−0.0003 (6)
C6	0.0224 (9)	0.0202 (9)	0.0266 (9)	−0.0051 (7)	−0.0034 (7)	0.0044 (7)
C7	0.0230 (9)	0.0162 (8)	0.0348 (10)	−0.0033 (7)	0.0087 (8)	−0.0008 (7)
C8	0.0174 (9)	0.0198 (9)	0.0320 (10)	−0.0012 (7)	0.0055 (7)	−0.0023 (7)
C9	0.0168 (8)	0.0207 (8)	0.0146 (8)	−0.0062 (7)	0.0003 (6)	−0.0039 (6)
C10	0.0217 (9)	0.0158 (8)	0.0245 (9)	−0.0048 (7)	0.0021 (7)	−0.0055 (7)
C11	0.0193 (8)	0.0175 (8)	0.0259 (9)	−0.0031 (7)	0.0036 (7)	−0.0083 (7)
C12	0.0181 (8)	0.0198 (8)	0.0153 (8)	−0.0055 (7)	−0.0024 (6)	−0.0015 (6)
C13	0.0198 (9)	0.0213 (9)	0.0178 (8)	−0.0049 (7)	0.0006 (6)	0.0003 (7)
C14	0.0171 (8)	0.0241 (9)	0.0146 (8)	−0.0054 (7)	0.0000 (6)	−0.0003 (6)
C15	0.0238 (9)	0.0215 (9)	0.0283 (9)	−0.0043 (7)	0.0040 (7)	−0.0039 (7)
C16	0.0206 (9)	0.0304 (10)	0.0290 (10)	−0.0037 (8)	0.0076 (7)	−0.0062 (8)
C17	0.0255 (9)	0.0246 (9)	0.0260 (9)	−0.0096 (8)	0.0052 (7)	−0.0056 (7)
C18	0.0216 (9)	0.0248 (9)	0.0229 (9)	−0.0054 (7)	0.0045 (7)	−0.0067 (7)

### Geometric parameters (Å, °)

**Table:**

Cd—S1	2.6379 (10)	C4—H4B	0.9900
Cd—S2	2.6626 (10)	C5—C6	1.527 (2)
Cd—N2	2.5403 (17)	C5—H5A	0.9900
Cd—S1^i^	2.6379 (10)	C5—H5B	0.9900
Cd—S2^i^	2.6626 (10)	C6—H6A	0.9800
Cd—N2^i^	2.5403 (17)	C6—H6B	0.9800
S1—C1	1.7369 (17)	C6—H6C	0.9800
S2—C1	1.7286 (18)	C7—C8	1.385 (2)
O1—C3	1.421 (2)	C7—H7	0.9500
O1—H1o	0.835 (10)	C8—C9	1.395 (2)
N1—C1	1.339 (2)	C8—H8	0.9500
N1—C2	1.475 (2)	C9—C10	1.390 (2)
N1—C4	1.479 (2)	C9—C12	1.471 (2)
N2—C11	1.336 (2)	C10—C11	1.386 (2)
N2—C7	1.347 (2)	C10—H10	0.9500
N3—C12	1.280 (2)	C11—H11	0.9500
N3—N4	1.418 (2)	C12—H12	0.9500
N4—C13	1.279 (2)	C13—C14	1.475 (2)
N5—C16	1.333 (3)	C13—H13	0.9500
N5—C17	1.344 (2)	C14—C15	1.391 (2)
C2—C3	1.516 (2)	C14—C18	1.393 (3)
C2—H2A	0.9900	C15—C16	1.388 (3)
C2—H2B	0.9900	C15—H15	0.9500
C3—H3A	0.9900	C16—H16	0.9500
C3—H3B	0.9900	C17—C18	1.385 (2)
C4—C5	1.521 (2)	C17—H17	0.9500
C4—H4A	0.9900	C18—H18	0.9500
			
N2^i^—Cd—N2	180	C4—C5—C6	110.58 (14)
N2^i^—Cd—S1	89.42 (4)	C4—C5—H5A	109.5
N2—Cd—S1	90.58 (4)	C6—C5—H5A	109.5
N2^i^—Cd—S1^i^	90.58 (4)	C4—C5—H5B	109.5
N2—Cd—S1^i^	89.42 (4)	C6—C5—H5B	109.5
S1—Cd—S1^i^	180	H5A—C5—H5B	108.1
N2^i^—Cd—S2	87.62 (4)	C5—C6—H6A	109.5
N2—Cd—S2	92.38 (4)	C5—C6—H6B	109.5
S1—Cd—S2	68.83 (3)	H6A—C6—H6B	109.5
S1^i^—Cd—S2	111.17 (3)	C5—C6—H6C	109.5
N2^i^—Cd—S2^i^	92.38 (4)	H6A—C6—H6C	109.5
N2—Cd—S2^i^	87.62 (4)	H6B—C6—H6C	109.5
S1—Cd—S2^i^	111.17 (3)	N2—C7—C8	123.52 (17)
S1^i^—Cd—S2^i^	68.83 (3)	N2—C7—H7	118.2
S2—Cd—S2^i^	180	C8—C7—H7	118.2
C1—S1—Cd	86.05 (6)	C7—C8—C9	119.02 (16)
C1—S2—Cd	85.43 (6)	C7—C8—H8	120.5
C3—O1—H1o	109.5 (16)	C9—C8—H8	120.5
C1—N1—C2	121.74 (13)	C10—C9—C8	117.67 (15)
C1—N1—C4	121.96 (13)	C10—C9—C12	118.88 (15)
C2—N1—C4	116.29 (13)	C8—C9—C12	123.44 (16)
C11—N2—C7	116.95 (15)	C11—C10—C9	119.30 (16)
C11—N2—Cd	119.88 (11)	C11—C10—H10	120.3
C7—N2—Cd	123.16 (11)	C9—C10—H10	120.3
C12—N3—N4	110.47 (14)	N2—C11—C10	123.54 (16)
C13—N4—N3	111.93 (14)	N2—C11—H11	118.2
C16—N5—C17	116.98 (16)	C10—C11—H11	118.2
N1—C1—S2	120.59 (12)	N3—C12—C9	121.48 (16)
N1—C1—S1	119.77 (12)	N3—C12—H12	119.3
S2—C1—S1	119.64 (10)	C9—C12—H12	119.3
N1—C2—C3	113.01 (13)	N4—C13—C14	119.59 (16)
N1—C2—H2A	109.0	N4—C13—H13	120.2
C3—C2—H2A	109.0	C14—C13—H13	120.2
N1—C2—H2B	109.0	C15—C14—C18	117.63 (16)
C3—C2—H2B	109.0	C15—C14—C13	120.38 (16)
H2A—C2—H2B	107.8	C18—C14—C13	121.99 (16)
O1—C3—C2	110.27 (14)	C16—C15—C14	118.89 (17)
O1—C3—H3A	109.6	C16—C15—H15	120.6
C2—C3—H3A	109.6	C14—C15—H15	120.6
O1—C3—H3B	109.6	N5—C16—C15	123.90 (17)
C2—C3—H3B	109.6	N5—C16—H16	118.0
H3A—C3—H3B	108.1	C15—C16—H16	118.0
N1—C4—C5	113.22 (13)	N5—C17—C18	123.21 (17)
N1—C4—H4A	108.9	N5—C17—H17	118.4
C5—C4—H4A	108.9	C18—C17—H17	118.4
N1—C4—H4B	108.9	C17—C18—C14	119.38 (17)
C5—C4—H4B	108.9	C17—C18—H18	120.3
H4A—C4—H4B	107.7	C14—C18—H18	120.3
			
N2^i^—Cd—S1—C1	−86.27 (7)	C4—N1—C2—C3	−87.99 (17)
N2—Cd—S1—C1	93.73 (7)	N1—C2—C3—O1	70.19 (17)
S1^i^—Cd—S1—C1	95 (100)	C1—N1—C4—C5	90.95 (18)
S2—Cd—S1—C1	1.40 (5)	C2—N1—C4—C5	−89.78 (16)
S2^i^—Cd—S1—C1	−178.60 (5)	N1—C4—C5—C6	175.96 (13)
N2^i^—Cd—S2—C1	88.89 (7)	C11—N2—C7—C8	0.3 (3)
N2—Cd—S2—C1	−91.11 (7)	Cd—N2—C7—C8	179.02 (14)
S1—Cd—S2—C1	−1.41 (5)	N2—C7—C8—C9	−0.1 (3)
S1^i^—Cd—S2—C1	178.59 (5)	C7—C8—C9—C10	−0.3 (3)
S2^i^—Cd—S2—C1	−29 (100)	C7—C8—C9—C12	−179.20 (17)
N2^i^—Cd—N2—C11	−146 (100)	C8—C9—C10—C11	0.5 (2)
S1—Cd—N2—C11	−10.78 (13)	C12—C9—C10—C11	179.47 (15)
S1^i^—Cd—N2—C11	169.22 (13)	C7—N2—C11—C10	−0.1 (3)
S2—Cd—N2—C11	58.06 (13)	Cd—N2—C11—C10	−178.83 (13)
S2^i^—Cd—N2—C11	−121.94 (13)	C9—C10—C11—N2	−0.3 (3)
N2^i^—Cd—N2—C7	35 (100)	N4—N3—C12—C9	176.90 (14)
S1—Cd—N2—C7	170.58 (14)	C10—C9—C12—N3	173.70 (15)
S1^i^—Cd—N2—C7	−9.42 (14)	C8—C9—C12—N3	−7.4 (3)
S2—Cd—N2—C7	−120.58 (14)	N3—N4—C13—C14	179.61 (14)
S2^i^—Cd—N2—C7	59.42 (14)	N4—C13—C14—C15	169.03 (16)
C12—N3—N4—C13	−177.28 (14)	N4—C13—C14—C18	−10.7 (3)
C2—N1—C1—S2	−2.1 (2)	C18—C14—C15—C16	−1.1 (3)
C4—N1—C1—S2	177.14 (10)	C13—C14—C15—C16	179.14 (16)
C2—N1—C1—S1	177.52 (10)	C17—N5—C16—C15	0.6 (3)
C4—N1—C1—S1	−3.2 (2)	C14—C15—C16—N5	0.4 (3)
Cd—S2—C1—N1	−178.09 (12)	C16—N5—C17—C18	−0.8 (3)
Cd—S2—C1—S1	2.29 (8)	N5—C17—C18—C14	0.1 (3)
Cd—S1—C1—N1	178.07 (12)	C15—C14—C18—C17	0.9 (3)
Cd—S1—C1—S2	−2.31 (8)	C13—C14—C18—C17	−179.34 (16)
C1—N1—C2—C3	91.28 (18)		

Symmetry codes: (i) −*x*+1, −*y*+1, −*z*+1.

### Hydrogen-bond geometry (Å, °)

**Table:**

*D*—H···*A*	*D*—H	H···*A*	*D*···*A*	*D*—H···*A*
O1—H1o···N5^ii^	0.84 (2)	1.98 (2)	2.810 (2)	176 (2)
C10—H10···O1^iii^	0.95	2.55	3.480 (3)	168
C3—H3a···N4^iv^	0.99	2.61	3.369 (3)	134

Symmetry codes: (ii) *x*+2, *y*, *z*−1; (iii) −*x*+1, −*y*, −*z*+1; (iv) *x*+1, *y*, *z*−1.
